# Forming cytoophidia prolongs the half-life of CTP synthase

**DOI:** 10.1038/s41421-019-0098-6

**Published:** 2019-06-18

**Authors:** Zhe Sun, Ji-Long Liu

**Affiliations:** 1 School of Life Science and Technology, ShanghaiTech University, 230 Haike Road, 201210 Shanghai, China; 2 Department of Physiology, Anatomy, and Genetics, University of Oxford, South Parks Road, Oxford, OX1 3PT UK

**Keywords:** Cell biology, Post-translational modifications

Dear Editor,

Intracellular compartmentation of biological processes is fundamental for a cell to function. One method is to form membrane-bound organelles, such as the mitochondria, endoplasmic reticulum (ER), and lysosomes, which have been extensively studied in the past decades. However, recent studies suggest that cells also contain organelles lacking a delimiting membrane as liquid droplets in response to cellular stress, such as U bodies and purinosomes^1,2^. Phase separation has been recognized as one of the mechanisms for the dynamic organization of these membraneless organelles^3^. In addition to these droplet structures, membraneless large-scale filamentous structures have also been reported in various metabolic enzymes^4^.

Cytidine-5′-triphosphate (CTP) not only serves as the building blocks for RNA, but also participates in the phospholipid synthesis and protein sialylation^4^. The lowest cellular concentration of CTP among four nucleotides (UTP, ATP, GTP, and CTP) makes it be the rate-limiting molecule for nucleic acids synthesis and other CTP-dependent events. CTP synthase (CTPS) is the rate-limiting enzyme that catalyzes the ATP-dependent conversion of UTP to CTP. We and others previously observed that CTPS can be assembled into long membraneless filamentous structures termed cytoophidia in fruit fly, bacteria, yeast, and mammalian cells^4,5^. We recently reported the presence of cytoophidia in a variety of human cancer tissues, such as colon, liver, and prostate cancers as well^6^. Previous studies have established a link between cytoophidia and CTPS enzymatic activity^7–10^. However, whether cytoophidia could serve as a storage of CTPS and a buffering system to maintain cellular homeostasis and meet the high CTP requirement of fast-growing cells like cancer cells is still elusive.

To examine the effect of forming cytoophidia on the levels of CTPS protein, we first treated HEK-293T cells stably expressing mCTPS1-GFP with the glutamine analog, 6-diazo-5-oxo-l-norleucine (DON), which can effectively induce the formation of CTPS cytoophidia (Supplementary Fig. S1a). Our data showed that the protein levels of mCTPS1-GFP were significantly increased upon DON treatment, especially at 36 h (Fig. 1a, b). Meanwhile, no increase was observed in the messenger RNA (mRNA) levels of mCTPS1-GFP after DON treatment (Supplementary Fig. S1b). We recently identified a colon cancer cell line SW480, in which CTPS can assemble into cytoophidia without any treatments^11^. To further determine the effect of cytoophidia on CTPS protein expression levels, we generated H355A and R294D mutant mCTPS1 tagged with GFP. These two mutations could not form cytoophidia even under DON treatment (Supplementary Fig. S2a). The overall tetramer structure, which is necessary for CTPS activity, was not affected by the H355A or R294D mutation^7,9^. Structure modeling showed that H355 and R294 locate at the interface between two consecutive tetramers (Supplementary Fig. S2b, c). Reduced cytoophidia formation by R294D and H355A might be due to the weakening of the interaction between CTPS1 tetramers. Consistent with the previous study^9^, enzymatic activity assay in vitro showed no significant difference in the catalytic activity between R294D mutant and wild-type hCTPS1 (Supplementary Fig. S2d). We next expressed wild-type, H355A and R294D mutant mCTPS1-GFP in SW480 cells stably. As expected, wild-type mCTPS1-GFP assembled into cytoophidia in SW480 cells in normal condition, while H355A and R294D mutant mCTPS1-GFP showed cytoplasmic distribution and did not assemble into cytoophidia (Supplementary Fig. S3a). Real-time PCR results showed no significant differences in the mCTPS1-GFP mRNA levels of SW480 cells stably expressing wild-type, H355A or R294D mutant CTPS1 (Supplementary Fig. S3b, c). We then compared the mCTPS1-GFP protein levels of those SW480 cell lines. Western blotting data showed that the protein levels of H355A or R294D mutant mCTPS1 were about five times lower than that of wild-type mCTPS1 (Fig. 1c–f). We next determined whether the higher level of wild-type mCTPS1 than those of H355A or R294D mutant mCTPS1 is indeed due to the formation of cytoophidia. HeLa cells, in which CTPS cannot form filamentous structure under normal conditions, were stably transfected with wild-type, H355A and R294D mutant mCTPS1 and then the protein and mRNA levels of mCTPS1 were analyzed. Our results showed that there was no obvious difference in the protein levels of mCTPS1 in these three stable HeLa cell lines when the mRNA levels of mCTPS1 were similar (Supplementary Fig. S4a, b).

**Fig. 1:**
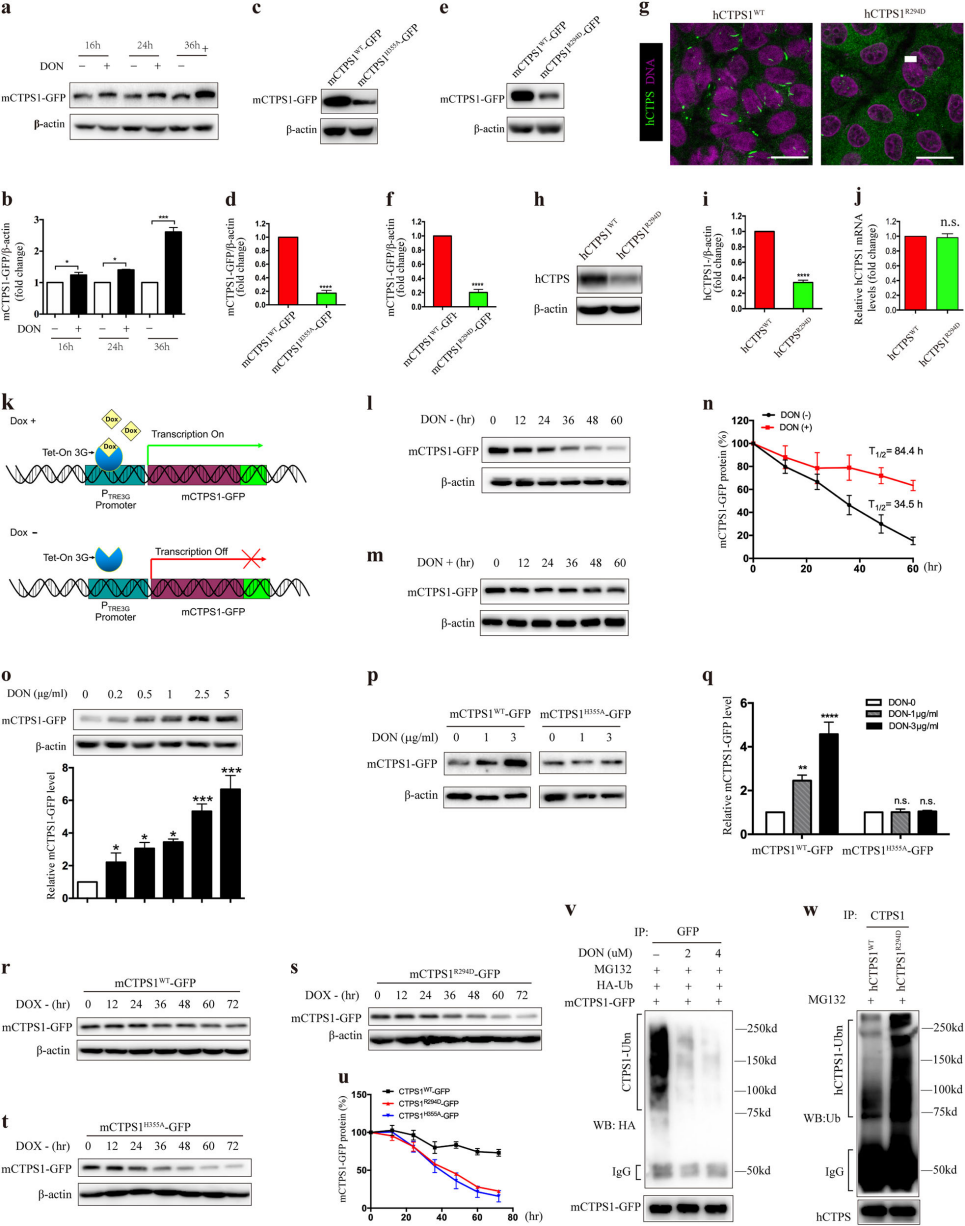
Forming cytoophidia prolongs the half-life of CTP synthase. **a** HEK-293T cells stably expressing mCTPS1-GFP were treated with 4 μg/ml DON for the indicated time, and cell lysates were prepared and analyzed by immunoblotting with anti-GFP antibody. **b** Quantitative data of mCTPS1-GFP protein level in **a**. **c**, **e** SW480 cells stably expressing mCTPS1^WT^-GFP, mCTPS1H^355A^-GFP (**c**) or mCTPS1^R294D^-GFP (**e**) were cultured for 3 days, and then subjected to western blotting analysis with anti-GFP antibody. **d**, **f** Quantitative data of mCTPS1-GFP protein level in **c** and **e**, respectively. **g–i** SW480 cells expressing endogenous wild-type CTPS1 (CTPS1^WT^) and those expressing R294D mutant CTPS1 (CTPS1^R294D^) were cultured for 3 days, followed by immunostaining (**g**) or western blotting against CTPS1 (**h**). **i** Quantitative data of CTPS1 protein levels in **h**. **j** The CTPS1 mRNA levels of SW480 CTPS1^WT^ and SW480 CTPS1^R294D+/−^ cells were analyzed by quantitative reverse transcription PCR (qRT-PCR). **k** Schematic diagram of TRE3G Tet-On system. **l**, **m** HEK-293T cells stably expressing Tet-On mCTPS1-GFP were cultured in the medium containing doxycycline (200 ng/ml) for 24 h, followed by culturing with the doxycycline-free medium without (**l**) or with (**m**) DON (4 μg/ml) for the indicated time. Lysates were prepared and analyzed by immunoblotting with appropriate antibodies. **n** Quantitative data of mCTPS1-GFP protein levels in **l** and **m**. **o** HEK-293T cells stably expressing Tet-On mCTPS1-GFP were cultured in the medium containing doxycycline (200 ng/ml) for 24 h, followed by culturing with the doxycycline-free medium with the indicated concentration of DON for 48 h. Lysates were prepared and analyzed by immunoblotting with appropriate antibodies. **p** HEK-293T cells stably expressing Tet-On mCTPS1^WT^-GFP (left three lanes) or Tet-On mCTPS1^H355A^-GFP (right three lanes) were treated with doxycycline (200 ng/ml) for 24 h, followed by culturing with doxycycline-free medium with the indicated concentration of DON for 48 h. Anti-GFP antibody was used to detect mCTPS1-GFP levels. **q** Quantitative data of mCTPS1-GFP protein levels in **p**. **r–t** SW480 cells stably expressing Tet-On mCTPS1^WT^-GFP (**r**), mCTPS1^R294D^-GFP (**s**) or mCTPS1^H355A^-GFP (**t**) were grown in the medium containing doxycycline (200 ng/ml) for 24 h, followed by culturing with the doxycycline-free medium for the indicated time. Lysates were prepared and subjected to western blotting analysis with anti-GFP antibody. **u** Quantitative data of mCTPS1-GFP protein levels in **r**–**t**. **v** HEK-293T cells stably expressing mCTPS1-GFP were transfected with HA-Ubiquitin, and then treated with the indicated concentration of DON for 36 h. MG132 (20 μM) was added during the last 16 h of treatment. Lysates prepared were subjected to immunoprecipitation by anti-GFP antibody. Immunoprecipitates were analyzed by immunoblotting using anti-HA antibody. **w** Wild-type and CTPS1 R294D mutant SW480 cells were treated with MG132 for 16 h. Lysates were prepared and subjected to immunoprecipitation by anti-CTPS1 antibody. Immunoprecipitates were analyzed by immunoblotting using anti-ubiquitin antibody. Mean ± S.E.M, *P < 0.05; **P < 0.01; ***P < 0.001; ****P < 0.0001 versus control. Scale bars = 20 μm. One of three to five similar experiments is shown. DAPI, 40,6-diamidino-2-phenylindole; DON, 6-diazo-5-oxo-l-norleucine

We further generated a heterozygous CTPS1 R294D mutant SW480 cell line (SW480 CTPS1^R294D+/−^) to determine whether disrupting cytoophidia could reduce endogenous CTPS protein levels. As shown in Fig. 1g, CTPS cytoophidia were hardly detectable in SW480 CTPS1^R294D+/−^ cells, suggesting that CTPS1^R294D^ can serve as a dominant-negative CTPS1 mutation. Western blotting data showed that the protein level of CTPS was much lower in SW480 CTPS1^R294D+/−^ cells as compared with that of SW480 CTPS1^WT^ cells (Fig. 1h, i). Moreover, no difference in the CTPS1 mRNA levels was observed in these two cell lines (Fig. 1j). Altogether, these results indicate that the promotion of cytoophidia assembly increases CTPS protein levels, while disruption of cytoophidia decreases CTPS protein levels.

We hypothesized that cytoophidia regulated CTPS protein levels through the prolongation of the half-life of CTPS protein. To test this idea, a Tet-On 3G tetracycline-inducible gene expression system was used to control exogenous mCTPS1 gene expression. In this system, the transcription of mCTPS1 was initiated by doxycycline (Dox) treatment, while the transcription will be stopped in the absence of Dox (Fig. 1k). Dox was added to the HEK-293T cells stably expressing Tet-On mCTPS1-GFP for 24 h prior to vehicle (Fig. 1l) or DON (Fig. 1m) treatment for the indicated time. Our results showed that DON-treated cells showed longer half-lives of mCTPS1-GFP than vehicle-treated cells (Fig. 1l–n). A similar result was also observed in the Tet-Off system. In contrast to the Tet-On system, the transcription of mCTPS1 was turned off by Dox treatment. Our data showed that DON treatment can effectively slow the degradation of mCTPS1-GFP as compared with vehicle treatment (Supplementary Fig. S5a, b). The dose-course study also demonstrated that treatment of HEK-293T cells expressing Tet-On mCTPS1-GFP with the indicated concentration of DON for 48 h after Dox deprivation significantly increased the protein levels of mCTPS1-GFP (Fig. 1o). We next assessed whether the accumulation of CTPS1 upon DON treatment is dependent on cytoophidia assembly by introducing an mCTPS1 mutation mCTPS1^H355A^, which lacks the ability to form cytoophidia under DON treatment (Supplementary Figs. S2a and 6a). As shown in Fig. 1p, q, DON treatment dramatically preserved the wild-type mCTPS1-GFP (mCTPS1^WT^-GFP) after Dox deprivation, while no difference was observed in mCTPS1^H355A^-GFP protein levels after DON treatment in HEK-293T cells. Similar results were also observed in HeLa cells (Supplementary Fig. S6b, c), suggesting that DON treatment prolongs CTPS1 half-life through promoting cytoophidia assembly.

To further confirm the role of cytoophida in the regulation of CTPS half-life, SW480 cells stably expressing Tet-On mCTPS1^WT^-GFP, Tet-On mCTPS1^H355A^-GFP and Tet-On mCTPS1^R294D^-GFP were treated with Dox for 24 h to initiate mCTPS1-GFP protein expression, followed by culturing with the Dox-free medium for the indicated time. Wild-type mCTPS1 showed longer half-life as compared with that of H355A and R294D mutant mCTPS1 (Fig. 1r–u). Thus, forming cytoophidia prolongs the half-life of CTPS.

The ubiquitin-proteasome system is the major pathway for the degradation of intracellular proteins. There are at least 18 potential ubiquitination sites of CTPS1 have been identified by proteomic mass spectrometry. We next determined the effect of cytoophidia assembly on CTPS ubiquitination. Our data showed a dramatic decrease in the ubiquitin modification of mCTPS1-GFP upon DON treatment (Fig. 1v). In addition, the ubiquitin modification of endogenous CTPS was also increased in SW480 CTPS1^R294D+/−^ cells as compared with wild-type SW480 cells (Fig. 1w). These data suggest that forming cytoophidia blocks the ubiquitin modification of CTPS. Thus, forming cytoophidia prolongs the half-life of CTPS likely through masking certain ubiquitination sites of CTPS1 and further suppresses proteasome-mediated CTPS1 degradation.

Altogether, a significant finding presented here is the demonstration that forming cytoophidia inhibits CTPS ubiquitination and further prolongs the half-life of CTPS. Therefore cytoophidia can serve as a storage of CTPS. The correlation between CTPS enzymatic activity and filamentation has been studied in different organisms^7–9,12^. For human CTPS, multiple lines of in vivo evidence from cell biological studies showed that the formation of cytoophidia could sequester CTPS enzyme activity. For example, we previously found that overexpression of CTPS dramatically promoted CTPS cytoophidia assembly in 293T cells. However, the intracellular CTP concentration only increased moderately^10^. A recent study also showed that the formation of cytoophidia under the condition of nutrient starvation reduced its enzymatic activity^9^. Thus, cytoophidia could function as a storage to protect CTPS from degradation so that the cell can harbor many CTPS protein molecules without releasing their activity and react to environmental changes rapidly. Nevertheless, the elevated enzymatic activity of human CTPS in cytoophidia has also been demonstrated in vitro assembly in the presence of substrates^6^. In this case, cytoophidia may preserve these active CTPS molecules in this large-scale filamentous structure to meet the high CTP requirement in fast-growing cells like cancer cells.

The protein expression and catalytic activity of CTPS are elevated in a variety of human cancers^13,14^. Importantly, knocking down of CTPS or suppressing CTPS enzymatic activity can effectively induce a decrease in cancer cell proliferation and increase the sensitivity of cancer cells to chemotherapies^14,15^. In fact, CTPS has been an attractive anti-cancer target for decades. However, the unwanted side effects like neurotoxicity, nausea and vomiting after CTPS inhibitors treatment hindered their further development^16,17^. Previous studies showed that the size and abundance of cytoophidia are positively related to the activity and expression of proto-oncogenes, including Myc, Casitas B-lineage lymphoma (Cbl), and activated cdc42-associated kinase (Ack) in *Drosophila*^12,18,19^. Our recent study has demonstrated the presence of CTPS cytoophidia in various human cancer tissues^6^. Thus, it may be of interest to investigate the role of cytoophidia in cancer development and chemotherapy drug resistance, and to further design small molecules to disrupt cytoophidia assembly may represent a new strategy for combating cytoophida-positive cancers.

Mounting evidence indicates that CTPS is not the only component of these filaments. We previously reported that inosine monophosphate dehydrogenase (IMPDH), the rate-limiting enzyme in de novo GTP biosynthesis, and asparagine synthetase (ASNS), an essential enzyme for biosynthesis of asparagine, can be assembled into similar filaments adjacent to the CTPS cytoophidium^20,21^. It may be that CTPS cytoophidia serves as a metabolic stabilizer and a cooperative platform for CTPS and many other metabolic enzymes. Different enzymes might coordinate with each other through forming cytoophidia. In the future, it seems interesting to demonstrate whether other metabolic enzymes can also be stored in this large-scale filamentous structure.

## Supplementary information

Supplementary Information

## Supplementary information

**Supplementary Information** accompanies the paper at (10.1038/s41421-019-0098-6).
