# Supplementary material for: Forming cytoophidia prolongs the half-life of CTP synthase
Source: Cell Discov. 2019 Jun 18;5:32. doi: 10.1038/s41421-019-0098-6 (PMC6579761; doi:10.1038/s41421-019-0098-6)
Supplement: Supplementary file 1 — Supplementary Information [file 41421_2019_98_MOESM1_ESM.pdf]

## Supplementary Information

## Materials and methods

### Regents and antibodies

Antibodies for CTPS (15914-1-AP) and ubiquitin (10201-2-AP) were purchased from ProteinTech. Antibody for GFP (11 814 460 001) was purchased from Roche. Antibodies for β-actin (ab6276) was purchased from Abcam. Antibody for HA (sc-7392) and Protein A/G-Agarose (sc-2003) was from Santa Cruz Biotechnology. Doxycycline (ab141091) was purchased from Abcam. 6-Diazo-5-oxo-L-norleucine crystalline (D2141-5MG) was purchased from Sigma. MG132 (S2619) was from Selleck Chemicals.

### Purification of hCTPS1 and activity assay

The human CTPS1 was cloned as C-terminal His-tagged fusion protein in pET28 expression vector and overexpressed in

*Escherichia coli* BL21 DE3 strain. In brief, cells were harvested and sonicated, lysates were clarified by centrifugation at 15,000 rpm for 30 min at 4°C. Clarified lysates were incubated with the His60 Ni Superflow resin, and then washed and eluted with imidazole. The elute was collected and concentrated by centrifugation in a 3kDa cut-off centrifugal filter unite (Millipore) for subsequent activity assay. The CTPS activity assay was conducted as described by Lynch *et al*., 2017 (ref. 6).

**Cell culture**

HEK-293T, HeLa and SW480 cells were cultured in Dulbecco's modified Eagle's medium (DMEM, SH30022.01, Hyclone), supplemented with 10% fetal bovine serum (04-001, Biological Industries) and antibiotics (100 U/ml penicillin and 100 mg/ml streptomycin, SV30010, Hyclone), in a humidified atmosphere containing 5% $CO_2$ at 37°C (normal culture conditions). Cell

transfections were carried out by using R0531 (Thermo Fisher Scientific) according to the manufacturer's instructions.

## Lentivirus-mediated stable cell line construction

Wild-type or mutant mCTPS1 with a GFP tag at C-terminal were cloned into Lenti-X™ Tet-On® 3G. Plasmids were co-transfected together with psPAX2 and pMD2.G into HEK 293T cells. Virus were collected at 48 hours after transfection. Cells were infected with appropriate lentiviruses in the presence of 8 μg/ml polybrene (Millipore) for 48 hours and then cultured in normal medium containing 0.5 μg/ml puromycin for 1 week. The resulting puromycin-resistant cells were used for further analysis.

## Establishment and characterisation of mutant cell colonies with CRISPR/Cas9

The plasmid pU6-(BbsI) CBh-Cas9-T2A-mCherry (Addgene plasmid #64324) was used for generating the CTPS1/R294D mutant SW480 cell line. Briefly, the plasmid was digested with BpiI (Thermo Fisher Scientific) and purified by PCR clean-up kit (AP-PCR-250G, Axygen). Subsequently, annealed sgRNA oligonucleotide was inserted into the digested plasmid with T4 DNA ligase (M0202S, New England Biolabs). Sequence of sgRNA is 5’-ttgatagatatgatcgcttgc-3’, and the sequence of homology-directed repair template is 5’-tagctattttggtctcatgatagcgtgtaccttctgagtaattgggtttttcttgacgtttatttgatag atatgatgacctat tggagacctgctctattgcccttgtgggcaaataca-3’. The resulted plasmid was transfected into SW480 cells, and the cells expressing mCherry were sorted with flow cytometer (BD Biosciences) and single cells were seeded into 96-well plate. Two weeks later, the genomic DNA was extracted and the modified region was amplified with primers shown below: forward primer, 5’-

GGGCGTGAGCTCATCCTG-3'; reverse primer, 5'-CTCAAAGCCACGAAGCAACC-3'. The PCR products were sequenced for the characterization of these cell lines.

## Immunoblotting

Total proteins were prepared with NP-40 lysis buffer [150 mmol/L NaCl, 1.0% NP-40, 50 mmol/L Tris (pH 8.0)], and then quantified with a BCA assay (Pierce). Lysates were separated by SDS-PAGE, and transferred to PVDF membranes (Roche). The membranes were then blocked with 5% nonfat milk in TBST for 1 hour at room temperature, followed by incubation with appropriate primary antibodies at 4°C overnight. After incubation with HRP-conjugated secondary antibody at room temperature for 1 hour, the signals of secondary antibodies were detected by an enhanced chemiluminescence system.

**Immunofluorescence**

Cells cultured on glass slides were fixed with 4% paraformaldehyde in PBS for 10 min, and then permeabilized with 0.2% Triton X-100 for 10 min. After blocking with 5mg/ml bovine serum albumin in PBS for 1 hour, cells were incubated with anti-CTPS antibodies overnight at 4°C. After the primary antibody reaction, samples were washed and incubated with FITC-labeled secondary antibodies for 1 hour. Finally, nuclear DNA were stained with 4, 6-diamino-2-phenylindole (DAPI) (Beyotime, Shanghai, China) for 5 min. The images were taken under a confocal laser scanning microscope (Zeiss 710).

**RNA extraction and quantitative Real-time PCR**

Total RNAs were extracted by Trizol (ER501-01; TransGen). The first-strand cDNA synthesis was conducted with PrimeScriptTMRT

Master Mix (RR0361; Takara). qRT-PCR reactions were performed using SYBR Green dye (B21703; Bimake) and the Applied Biosystems 7500 Fast Real-Time PCR System. Primers used for GFP (forward primer, 5'- AAGCTGACCCTGAAGTTCATCTGC -3'; reverse primer, 5'- GTCTTGTAGTTGCCGTCGTCCTTGAA -3'), CTPS1 (forward primer, 5'- CAGTGTGGGCACAATACTCAA -3'; reverse primer, 5'- CGCTCATAGTTACCCAGGTCA -3') and β-actin (forward primer, 5'-CATGTACGTTGCTATCCAGGC-3'; reverse primer, 5'- CTCCTTAATGTCACGCACGAT-3'). The resulting values were normalized to β-actin expression.

## Ubiquitination assays

For mCTPS1-GFP ubiquitination assays, HEK-293T cells stably expressing Tet-On mCTPS1-GFP were transfected with HA-Ubiquitin, and then treated with the indicated concentration of DON

for 36 hours in the presence of Doxycycline (200ng/ml). MG132 (20μM) was added during the last 16 hours of treatment. Lysates prepared were subjected to immunoprecipitation by anti-GFP antibody. Immunoprecipitates were analyzed by immunoblotting using anti-HA antibody. For endogenous CTPS ubiquitination assays, Wild-type and CTPS1 R294D mutant SW480 cells were treated with MG132 for 16 hours. Lysates were prepared and subjected to immunoprecipitation by anti-CTPS1 antibody. Immunoprecipitates were analyzed by immunoblotting using anti-ubiquitin antibody.

**Statistical analysis**

Two-tailed unpaired Student’s t-test was used for comparisons between two groups and ordinary one-way ANOVA with Tukey's multiple comparison post-test was used to compare variables among three or more groups. $P \leq 0.05$ was considered statistically

significant. All analyses were performed using GraphPad Prism version 6.00 (GraphPad Software, San Diego, CA, USA, www.graphpad.com).

**Supplementary figures 1-6 and figure legends**

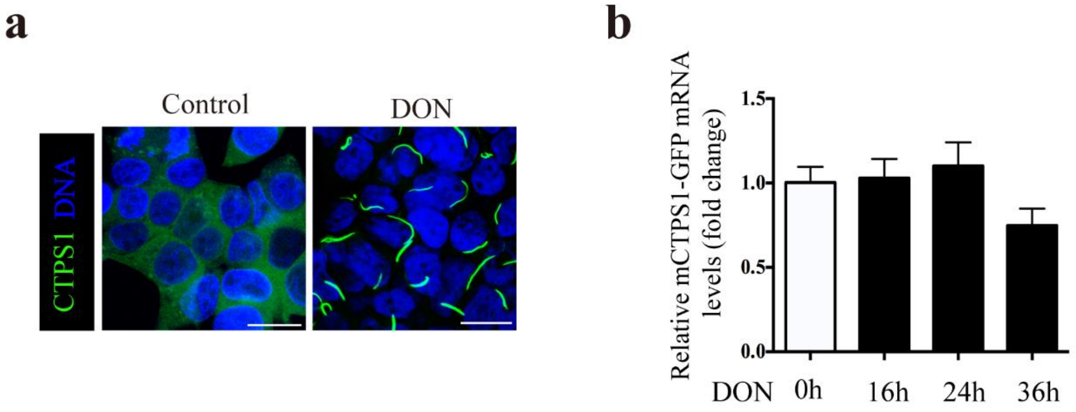


**Fig. S1. DON treatment does not increase mCTPS1-GFP mRNA levels.**

**(a)** HEK-293T cells stably expressing mCTPS1$^{WT}$-GFP were treated with DON (4μg/ml) for 24 hours, the cytoophidia assembly was analyzed by confocal microscope. **(b)** HEK-293T cells stably expressing mCTPS1-GFP were treated with 4μg/ml DON for the indicated time, and then subjected to qRT-PCR analysis for the mRNA expression of mCTPS1-GFP. Scale bars = 20 μm. One of three similar experiments is shown. DON, 6-diazo-5-oxo-L-norleucine.

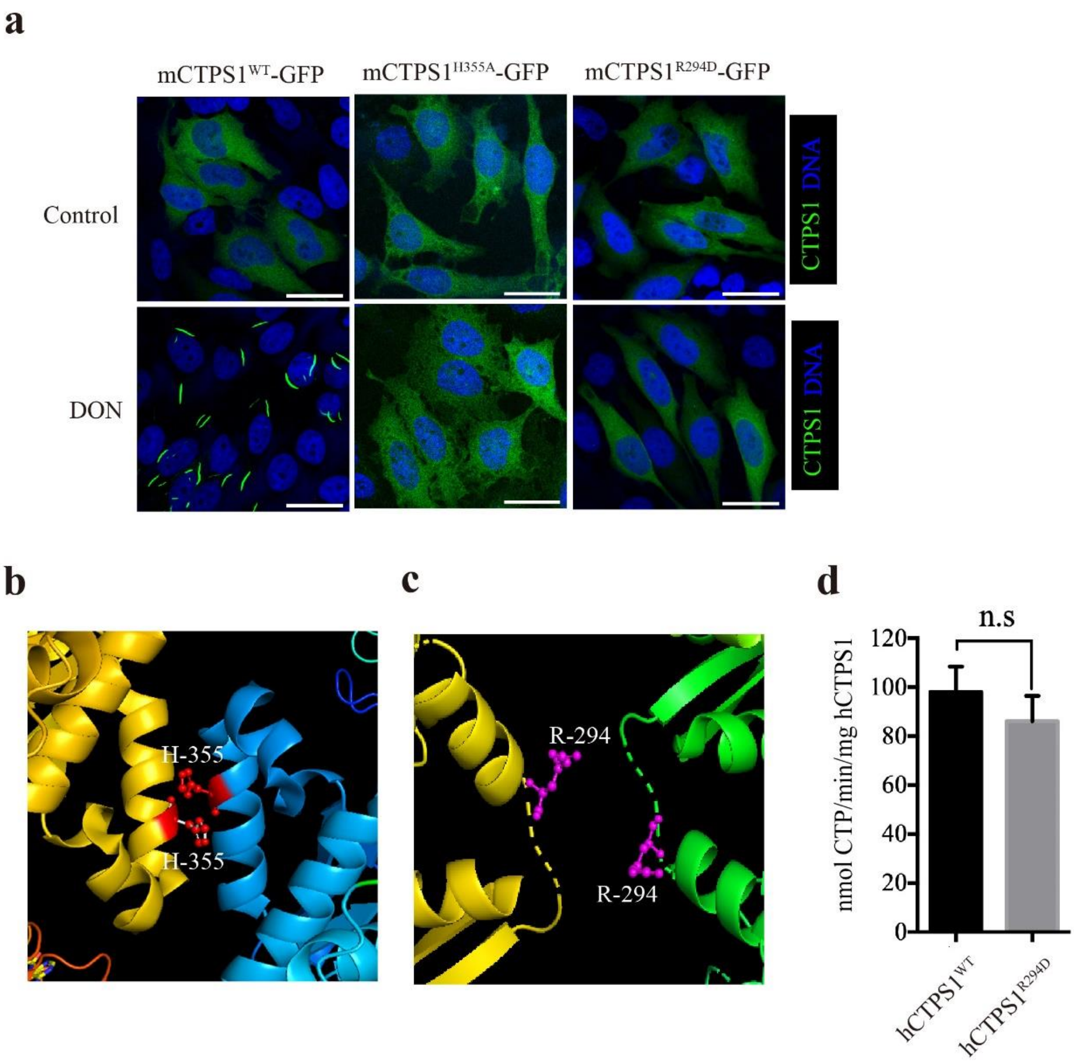


**Fig. S2. mCTPS1$^{H355A}$ and mCTPS1$^{R294D}$ are failed to form cytoophidia under DON treatment**

**(a)** HeLa cells transfected with mCTPS1$^{WT}$-GFP, mCTPS1$^{H355A}$-GFP or mCTPS1$^{R294D}$-GFP were treated with vehicle or DON (4μg/ml) for 6 hours, the cytoophidia assembly was analyzed by confocal microscope. **(b, c)** Structure modeling showed that H355 and R294

locate at the interface between two consecutive tetramers. **(d)** There is no significant difference in the enzymatic activity between wild type and R294D mutant hCTPS1. Scale bars = 20 μm. One of three similar experiments is shown. DON, 6-diazo-5-oxo-L- norleucine.

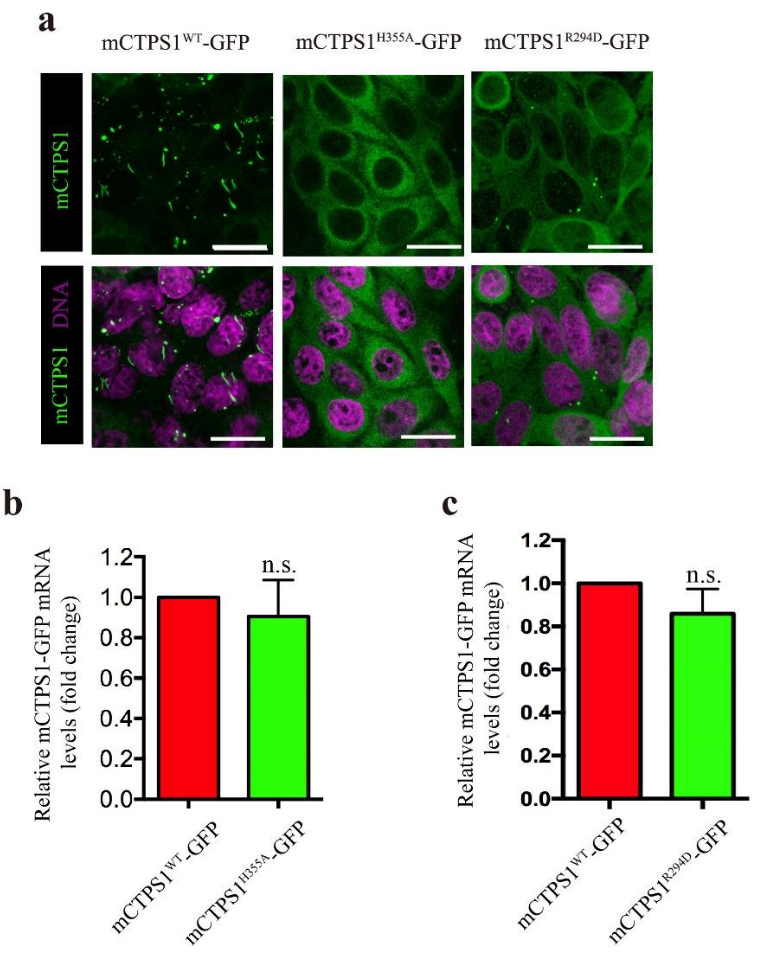


**Fig. S3. Forming cytoophidia does not change CTPS mRNA levels**

**(a)** SW480 cells stably expressing mCTPS1$^{WT}$-GFP, mCTPS1$^{H355A}$-GFP or mCTPS1$^{R294D}$-GFP were cultured for 3 days before fixation, cytoophidia assembly was analyzed by confocal microscope. Nuclei were labeled with DAPI (magenta). **(b, c)** SW480 cells stably expressing mCTPS1$^{WT}$-GFP, mCTPS1$^{H355A}$-GFP or mCTPS1$^{R294D}$-

GFP were cultured for 3 days in the presence of Doxycycline, and then subjected to qRT-PCR analysis for the mRNA expression of mCTPS1-GFP. One of three similar experiments is shown. Mean ± S.E.M, n.s. = not significant.

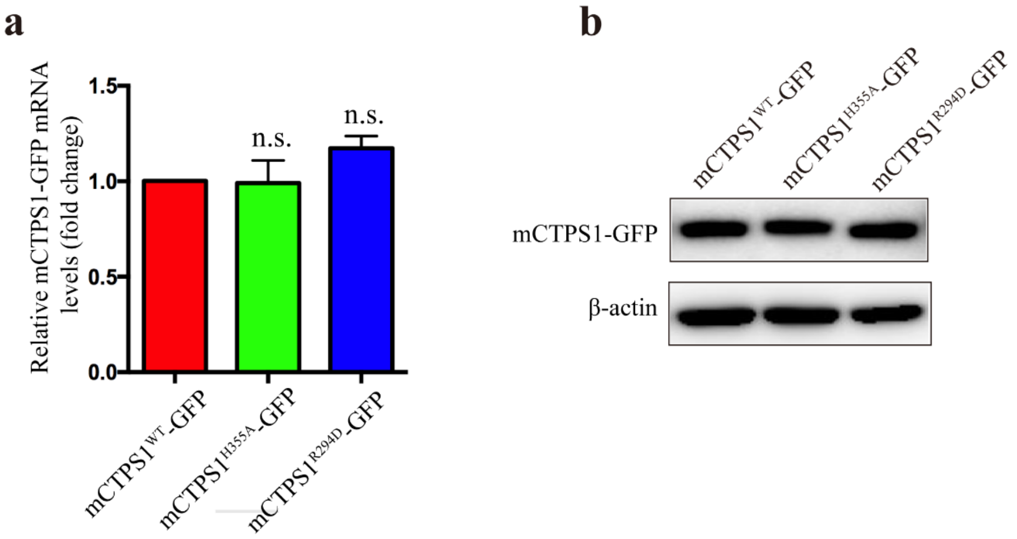


**Fig. S4. mCTPS1$^{H355A}$ and mCTPS1$^{R294D}$ mutation do not change mCTPS1 protein levels in HeLa cells**

**(a, b)** HeLa cells stably expressing mCTPS1$^{WT}$-GFP, mCTPS1$^{H355A}$-GFP or mCTPS1$^{R294D}$-GFP were cultured for 2 days, and then subjected to qRT-PCR analysis for the mRNA expression of mCTPS1-GFP **(a)**, or subjected to immunoblotting analysis with anti-GFP antibody **(b)**. One of three similar experiments is shown. Mean ± S.E.M, n.s. = not significant.

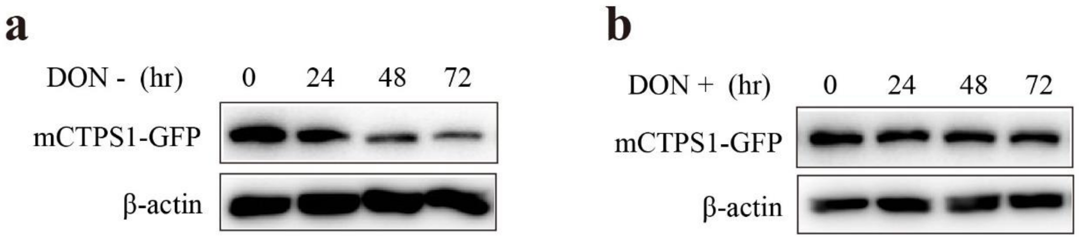


**Fig. S5. DON treatment slows mCTPS1-GFP protein degradation.**

**(a, b)** HEK-293T cells stably expressing Tet-Off mCTPS1-GFP were cultured in the medium without Doxycycline for 24 hours, followed by culturing with the medium containing Doxycycline (200ng/ml) without **(a)** or with **(b)** DON (4μg/ml) for the indicated time. Lysates were prepared and analyzed by immunoblotting with appropriate antibodies. One of three similar experiments is shown. DON, 6-diazo-5-oxo-L- norleucine.

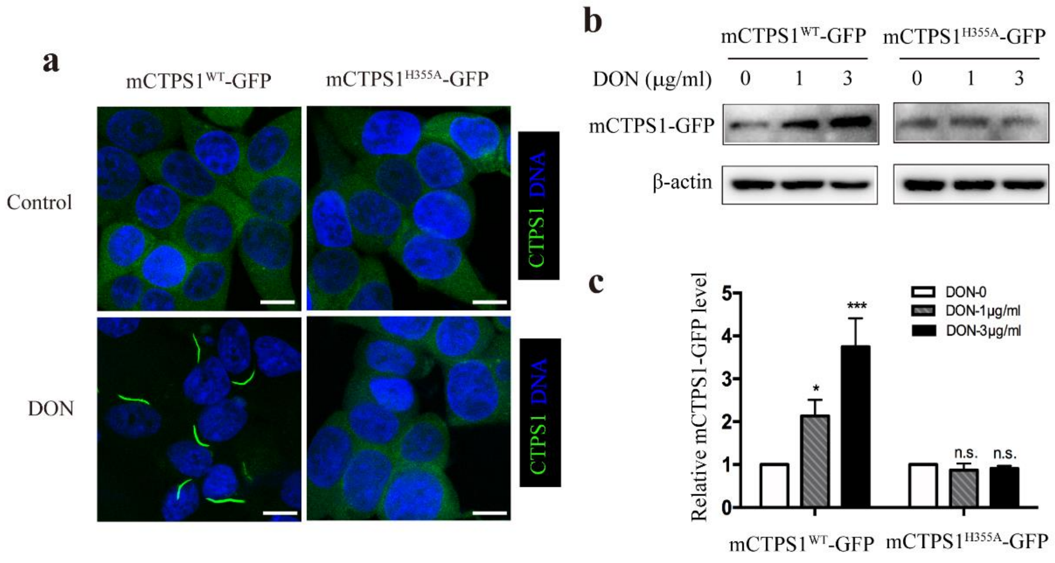


**Fig. S6. DON inhibits CTPS degradation depends on the formation of cytoophidia.**

**(a)** HEK-293T cells stably expressing Tet-On mCTPS1$^{WT}$-GFP or Tet-On mCTPS1$^{H355A}$-GFP were treated with Doxycycline (200ng/ml) and DON (4μg/ml) for 24 hours, the cytoophidia assembly was analyzed by confocal microscope. **(b)** HeLa cells stably expressing Tet-On mCTPS1$^{WT}$-GFP (left three lanes) or Tet-On mCTPS1$^{H355A}$-GFP (right three lanes) were treated with Doxycycline (200ng/ml) for 24 hours, followed by culturing with Doxycycline-free medium with the indicated concentration of DON for 48 hours. Anti-GFP antibody was used to detect mCTPS1-GFP expression. **(c)** Quantitative data

of mCTPS1-GFP protein expression in **(b).** Mean ± S.E.M, n.s. = not significant. *P<0.05; ***P<0.001; Scale bars = 10 μm One of three similar experiments is shown. DON, 6-diazo-5-oxo-L- norleucine.
